# Best of Both Worlds: Simultaneous High-Light and Shade-Tolerance Adaptations within Individual Leaves of the Living Stone *Lithops aucampiae*

**DOI:** 10.1371/journal.pone.0075671

**Published:** 2013-10-23

**Authors:** Katie J. Field, Rachel George, Brian Fearn, W. Paul Quick, Matthew P. Davey

**Affiliations:** 1 Animal and Plant Sciences, Western Bank, University of Sheffield, Sheffield, United Kingdom; 2 Abbey Brook Cactus Nursery, Matlock, Derbyshire, United Kingdom; 3 Department of Plant Sciences, Downing Street, University of Cambridge, Cambridge, United Kingdom; Mount Allison University, Canada

## Abstract

“Living stones” (*Lithops spp.*) display some of the most extreme morphological and physiological adaptations in the plant kingdom to tolerate the xeric environments in which they grow. The physiological mechanisms that optimise the photosynthetic processes of *Lithops* spp. while minimising transpirational water loss in both above- and below-ground tissues remain unclear. Our experiments have shown unique simultaneous high-light and shade-tolerant adaptations within individual leaves of *Lithops aucampiae*. Leaf windows on the upper surfaces of the plant allow sunlight to penetrate to photosynthetic tissues within while sunlight-blocking flavonoid accumulation limits incoming solar radiation and aids screening of harmful UV radiation. Increased concentration of chlorophyll *a* and greater chlorophyll *a*∶*b* in above-ground regions of leaves enable maximum photosynthetic use of incoming light, while inverted conical epidermal cells, increased chlorophyll *b*, and reduced chlorophyll *a*∶*b* ensure maximum absorption and use of low light levels within the below-ground region of the leaf. High NPQ capacity affords physiological flexibility under variable natural light conditions. Our findings demonstrate unprecedented physiological flexibility in a xerophyte and further our understanding of plant responses and adaptations to extreme environments.

## Introduction

Xerophytes often display unusual morphological and physiological characteristics to tolerate the challenging abiotic conditions of their native environments. Few traits are more extreme than those that have been selected for in South African “living stones” (*Lithops* spp.) where the majority of their biomass, including much of their photosynthetic tissue, is underground [1]. Subterranean autotrophy seems at first counterintuitive, however, the stresses imposed by the xeric environment in which *Lithops* inhabit [2] can be mitigated by the cooler and more stable conditions in the soil. Despite this, the precise mechanisms by which *Lithops* maximises subterranean photosynthesis while minimising transpirational water loss remain unknown.

**Figure 1:**
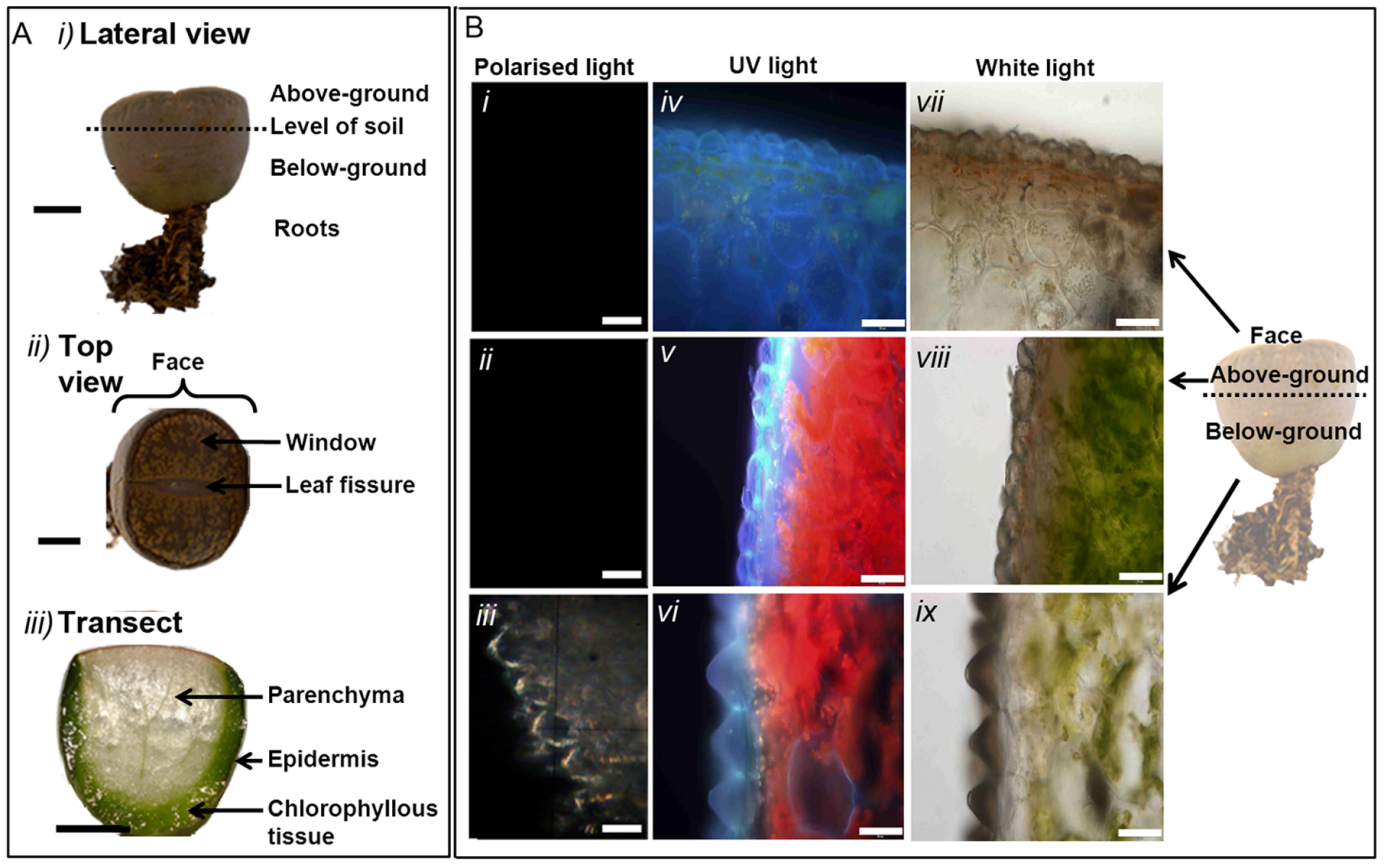
Anatomy of *Lithops aucampiae* A *i).* Un-earthed plant, lateral view; ***ii)*** Plant face and ***iii)*** vertical section through leaf transect. Bar  = 10 mm **B** longitudinal leaf transect showing epidermis under crossed-polarised light (***i, ii, iii***), UV (***iv, v, vi***) and white light (***vii, viii, ix***). Scale bar  = 50 µm.

*Lithops* is a member of the Aizoaceae, sub family Ruschiodiae [2]. The plant body consists of a very short stem with a pair of fused, succulent leaves (Fig. 1a *i*), resulting in low surface area to volume ratio. This allows *Lithops* to store substantial quantities of water per unit of exposed plant surface, enabling it to survive prolonged periods of drought [3]. The epidermis of many arid-dwelling plants is specialised for water conservation by reducing transpirational water loss [4], [5], optimising photosynthetic rate and regulating energy budgets [3]. This is taken to the extreme in *Lithops*; the top surface, or “face”, possesses “windows” of translucent epidermis [6] allowing light penetration to photosynthetic tissues deep within the subterranean leaf (Fig. 1(a) *i-iii*). Until recently it was thought the function of these windows was to enhance below-ground photosynthesis by allowing increased solar radiation to penetrate photosynthetic tissues, however it is now known that the presence of large epidermal windows can actually inhibit photosynthesis as a result of increased internal leaf temperatures through greater penetration of solar radiation [7]. An additional epidermal adaption of members of the Aizoaceae is the formation of enlarged, specialised epidermal cells. These occur throughout the plant kingdom, sometimes functioning as storage for various secondary metabolites and crystallised minerals [8], [9].

In common with other members of the Mesembryanthemaceae, *Lithops* employs a mode of photosynthesis known as Crassulacean Acid Metabolism (CAM) [5]. CAM reduces transpirational water loss while optimising photosynthetic rate by opening stomata for CO_2_ assimilation only at night when temperatures are cooler and the drop in water vapour concentration from leaf–to–air is minimal [3]. By fixing atmospheric CO_2_ into malate via oxaloacetate, CAM plants are able to store carbon at night for use in the Calvin cycle in daylight hours allowing stomata to be closed, thus preventing desiccation through excess transpiration [3].

Xerophytes are unavoidably over-exposed to solar irradiance, resulting in excess absorption of light energy beyond that which can be utilized effectively for photosynthesis [10]. In order to offset the inevitable excess of absorbed energy, plants utilise non-photochemical mechanisms that quench singlet-excited chlorophylls and dissipate the excess excitation energy as heat [11], a process known as non-photochemical quenching (NPQ). In plants with above-ground photosynthetic organs, the ability to dissipate inescapable excess solar radiation through NPQ is essential in maintaining optimal rates of photosynthesis while affording the plant protection against oxidative damage. The degree or significance of NPQ in plants with partially subterranean photosynthetic tissues is currently not well described in the literature.

In this study we examine the functional significance of physiological and morphological adaptations in the yellow-flowering, medium sized *Lithops aucampiae* (Fig. 1a) in relation to its photosynthetic processes using a unique combination of ecophysiological and metabolomics approaches. Specifically, we test the hypotheses that there is spatial heterogeneity in the photosynthetic adaptations of individual *L. aucampiae* leaves to both high light and shading; that regional differences exist in epidermal adaptations (windows, epidermal cells, pigmentation) and that these, combined with specialised regional physiologies (NPQ, photosynthetic pigmentation), enhance photosynthesis while minimising transpirational water loss under variable external light conditions. Our results provide novel insights into the specialised morphology and biochemistry of *L. aucampiae*, revealing unprecedented physiological flexibility in a xerophyte.

## Methods

*Lithops aucampiae* were selected from Abbey Brook *Lithops* UK national collection (Derbyshire, UK) and transferred to a controlled environment chamber (SGC2352/FM chamber, Sanyo-Gallenkamp, Japan), maintained under a day/night regime designed to mimic optimal growth conditions experienced in autumn/winter within their natural habitat. These conditions were 12/12 hour, 20/15°C, 550 μmol m^2^ s^−1^ irradiance, 50% RH, 440 ppm CO_2_. 60 nm leaf sections were viewed with an Olympus BX51 microscope (Olympus, Essex, UK) under white and UV fluorescence (UV excitation: 380–385 nm; emission: 420 nm) and with a cross-polarising light microscope (Swift, Basingstoke, UK) to visualise mineral deposition within epidermal cells/cell walls.

Tissue samples from the face and both the above- and below-ground regions of each leaf edge (3 tissue samples per region per plant, 3 plants; *n* = 3) surface (Fig. 1) were frozen in liquid nitrogen and homogenized. Owing to the morphology of *L. aucampiae,* the leaf edges comprise greater surface area and experience greater exposure to abiotic stresses than either the abaxial or adaxial leaf surfaces; therefore tissue sampling was conducted using these areas. Pigments were extracted using standard protocols (see Methods S1) and absorbance measured at 665 nm, 649 nm and 470 nm using a spectrophotometer (Lambda 40 UV/VIS, Perkin Elmer, Massachusetts, USA). Concentrations of chlorophyll *a, b* and carotenoids were calculated as in [12]. Steady-state quantum yield (Ф_PSII_) and NPQ of entire leaves (*n* = 36) were measured through chlorophyll fluorescence imaging (Technologica LTD, Colchester, UK). Actinic light levels of 100 and 500 µmol m^−2^ s^−1^ were applied prior to a further photosynthesis-saturating 3000 µmol m^−2^ s^−1^ PPFD pulse of 200 ms in duration in order to obtain measures of the operational efficiency of photosystem II (Ф_PSII_) and the levels of light dissipated as heat (NPQ). Epidermal tissue samples (3 leaf samples per plant, 3 individual plants; *n* = 3) from all leaf regions were analysed by HPLC for pigmentation. Bi-phasic metabolite extracts were prepared directly from frozen plant tissue (see SI). Both phases were analysed by HPLC (Hewlett Packard Series 1090 liquid chromatography system), full protocols are in SI.

Metabolite fingerprinting of plants sampled at 05∶30 (pre-dawn) and three at 17∶30 (pre-dusk) was carried out using Direct Injection Mass Spectrometry (SI) (3 samples per plant, 3 individual plants; *n* = 3) as in [13]. Metabolite profiles were compared by unsupervised Principal Component Analysis (PCA) using Simca-P+V12.0 (Umetrics, Sweden). Further details are available in SI. Treatment effects were analysed using ANOVA (Minitab v13, Minitab Inc., Pennsylvania, USA) following assumptions of a general linear model factorial design with post-hoc tests where appropriate (see SI).

## Results

### Epidermal anatomy and pigmentation

Longitudinal leaf sections viewed under both white and UV light show the epidermis of *L.aucampiae* consists of tightly packed cells (Fig. 1*b*). The transect images show the face and above-ground region of the plant to be covered in large, flattened cells while the below-ground region is made up of clearly defined inverted conical shaped epidermal cells (Fig. 1(b) *iv-ix*). When viewed under cross-polarised light, the conical below-ground epidermal cells showed internal microcrystalline deposits around or within cell walls with light inference properties corresponding to that of calcium oxalate [14] (Fig. 1(b) *iii*), previously observed in foliar tissues of other plant species [15], [16]. No reflectance of cross-polarised light was observed in any of the other sections (Fig. (b)*i,ii*).

**Figure 2:**
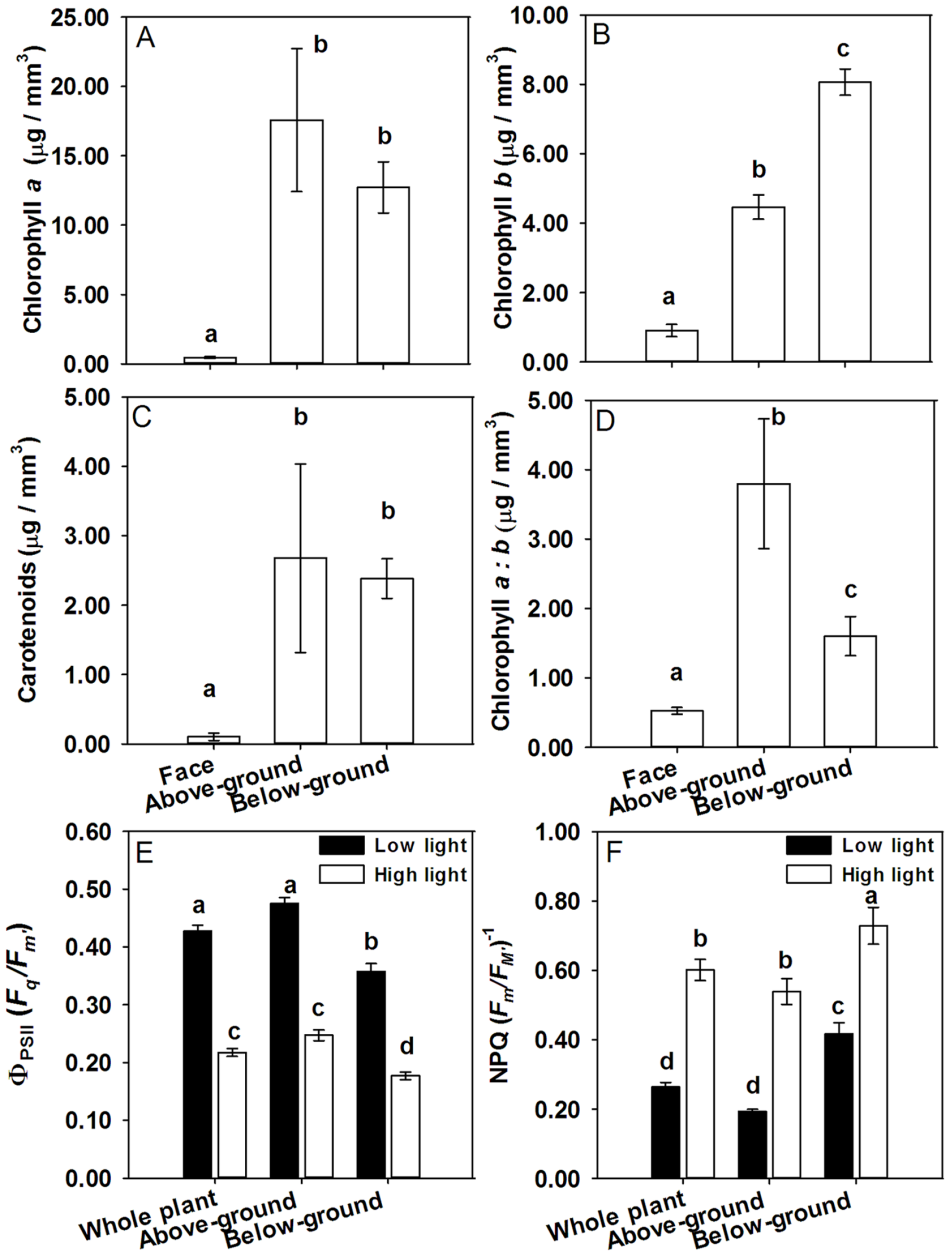
Photosynthetic physiology of *L. aucampiae*. Concentration of: **A** Chlorophyll a **B** Chlorophyll *b* and **C** Carotenoids, determined by HPLC. **D** chlorophyll a: b. n = 3±SE. **E** Operating efficiency of photosystem II (ФPSII) **F** Non-Photochemical Quenching (NPQ). n = 36,±SE. Bars sharing the same letter are not statistically different at P<0.05.

Chlorophyll *a* was the most abundant pigment in each region of *L. aucampiae*, followed by chlorophyll *b*, and carotenoid pigments being of lowest concentration (Fig. 2*a-c*). The face contained the lowest abundance of pigments in any of the regions measured. There was no significant difference in the concentration of carotenoids between the above- and below-ground regions (Fig. 2*c*).The chlorophyll *b* concentration of the below ground section was also significantly lower in the above-ground section. Chlorophyll *a*: chlorophyll *b* was significantly lower in the below-ground region than in the above-ground region (Fig. 2*d*).

### Photosynthetic activity in *L. aucampiae*

Optimised studies using plants acclimated to actinic light levels of 500 µmol m^−2^ s^−1^ and 100 µmol m^−2^ s^−1^ revealed that all regions of the plants exposed to the high light treatment had lower mean Ф_PSII_ values compared to those grown at lower actinic light (Fig. 2*e*; *F*_(1,215)_  = 289.11, *P*<0.001). Ф_PSII_ was significantly greater in the above-ground region than the below-ground region at each light level; however the difference between light levels was less marked in the below-ground region than in the above-ground region (Fig. 2*e*).

The opposite trend was true for NPQ with the high actinic light treatment producing the greatest mean NPQ values in all regions compared to the lower actinic treatment (F_(1,215)_  = 143.65, *P*<0.001). The highest mean NPQ value was observed in the below-ground region of the plant (Fig. 2*f*).

When plants were exposed to increasing actinic light levels, Ф_PSII_ decreased dramatically in the above-ground region (Fig. 2*e*). Ф_PSII_ also decreased in the below-ground region, although these values remained consistently lower than the values for the above-ground region at every actinic level measured (Fig. 2*e*). NPQ values had the opposite trend, as values increased with increasing actinic light levels (Fig. 2*f*).

### Metabolite profiling

**Figure 3:**
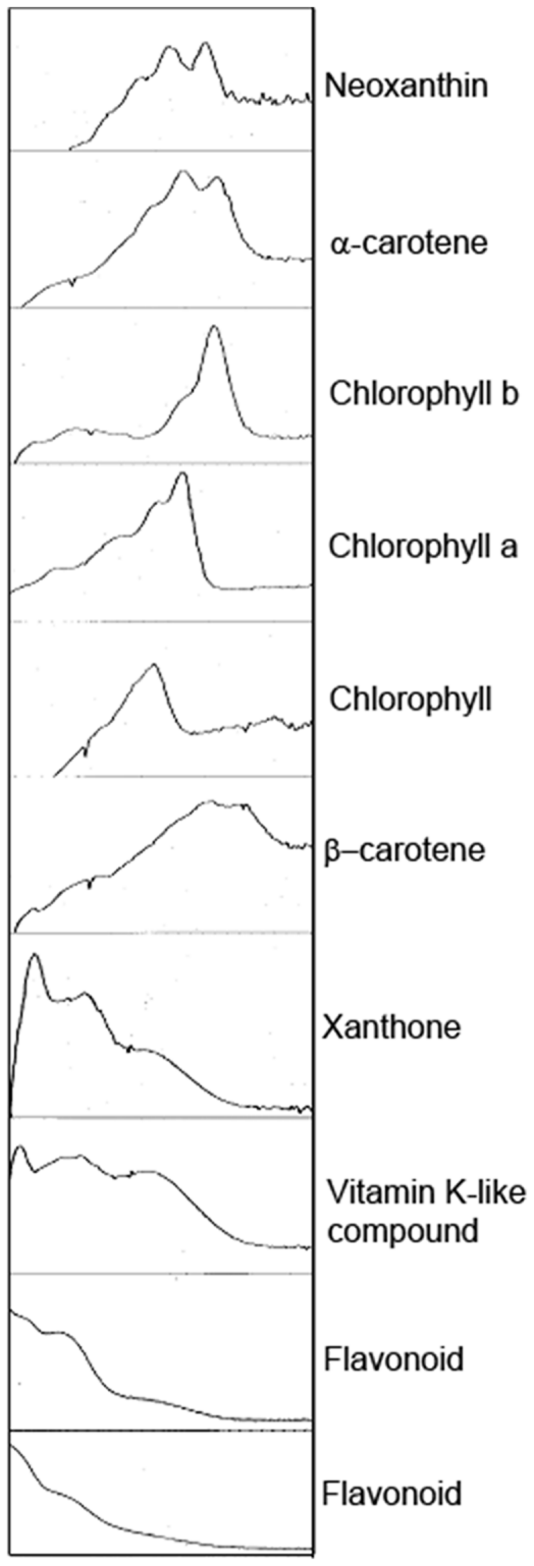
UV absorbance traces for metabolite identification. UV traces showing absorbance spectra and putative ID of metabolites obtained through HPLC-PDAD.

Metabolites were identified as photosynthetic pigments, being either chlorophylls (eluted after 19 min, 20 min and 21 min; Table 1, Fig. 3) or carotenoids (eluted after 8 min, 15 min and 22 min; Table 1, Fig. 3). UV-absorbing metabolites, likely to be anthocyanins, were identified in the above-ground region along with other non-photosynthetic pigments such as xanthone in the face (Table 1, Fig. 3).

**Table 1:** Metabolite identification by HPLC.

Plant section	Solvent phase	Retention time (min)	Putative compound	PDA UV λ_max_ (nm)
Whole plant	Organic	8	Neoxanthin	440, 465
Whole plant	Organic	15	α-carotene	445, 475
Whole plant	Organic	19	Chlorophyll *b*	345, 460
Whole plant	Organic	20	Chlorophyll *a*	385, 415, 430
Whole plant	Organic	21	Chlorophyll	410, 505
Whole plant	Organic	22	β-carotene	455, 485
Face	Aqueous	1	Xanthone	285, 330, 390
Face	Aqueous	2	Vitamin K_1_- like compound	275, 315, 370
Above-ground	Aqueous	2	Flavonoid	275, 305, 380
Above-ground	Aqueous	3	Flavonoid	270, 320

Metabolite fingerprinting of whole plants revealed clear metabolic differences between pre-dawn and pre-dusk samples. Principal Component Analysis (PCA) of the molecular ions detected within the aqueous phase showed distinct separation of the pre-dawn and pre-dusk samples along PC1 (Methods S1). Malate (133 *m/z*) was identified as a highly discriminatory compound between treatments and was found in greater abundance (530855.6 TIC) in pre-dawn samples compared to pre-dusk (289211.1 TIC) alongside many key tricarboxylic acid (TCA) cycle components (Table 2). This analysis also showed that carbohydrates, including hexose sugars, were of lower abundance in pre-dawn samples compared to those taken pre-dusk (Table 2).

**Table 2:** Putative metabolites in *L. aucampiae* sampled pre-dawn and pre-dusk (*n* = 3). *P<0.05, Student's T-test.

	Mean molecular mass (Da)	SE	Class of compound	Pre-dawn TIC	Pre-dusk TIC	Fold change	Sig.
Oxaloacetate	132.0	0.004	Organic acid	11669.8	5614.4	2.08	*
Citrate	192.0	0.006	Organic acid	328133.3	166400.0	1.97	*
Uracil	112.0	0.004	Nucleobase	244377.8	122427.8	1.99	*
Fumarate	116.0	0.004	Organic acid	466200.0	241944.4	1.93	*
Pyruvic acid	88.0	0.003	Organic acid	27568.9	52361.1	1.90	*
Malate	134.0	0.004	Organic acid	530855.6	289211.1	1.84	*
Succinate	118.0	0.004	Organic acid	10527.7	5702.0	1.84	*
cis-Aconitate	174.0	0.006	Organic acid	33801.1	19471.1	1.76	*
2-Oxoglutarate	146.0	0.005	Organic acid	945.9	1429.0	1.51	*
Phosphoenolpyruvate	167.9	0.005	Organic acid	2806.8	1870.3	1.50	*
Oxalosuccinic acid	190.0	0.006	Organic acid	1152.1	993.0	1.16	*
Hexose	342.1	0.011	Monosaccharide	17172.2	14943.3	1.15	*
Phenylpyruvate	164.0	0.005	Organic acid	55703.3	89098.9	0.63	*

## Discussion

Our research has demonstrated for the first time that *L. aucampiae* exhibits simultaneous high-light and shade-specific functional adaptations simultaneously within individual leaf structures. We hypothesise that this maintains optimal photosynthesis under the xeric conditions experienced in its native desert habitat.

Our anatomical observations of *L. aucampiae* using white-light and UV microscopy show *L. aucampiae* leaf structures to possess a highly varied epidermal structure, in common with others within the genera [17]. Cells on the face and above-ground regions of *L. aucampiae* leaves are large and flat, while those covering the below-ground foliar region are conical (Fig. 1*b*). Epidermal cells such as those in our observations are thought to have important functions in water storage, ion and pigment accumulation, light guiding and photosynthetic capacity [18]–[21]. It is possible the cone-shaped epidermal cells that cover the below-ground regions of the plant leaves serve to minimize loss of light and to maximise below-ground internal reflectance.

Calcium oxalate druses are often found within epidermal cells similar in structure to those we observed in *Lithops* in a diverse array of plant species including *Dieffenbachia seguine* [9], *Aesculus hippocastanum* [22] and, notably, more than 80 *Conophytum* species [23], themselves being close relatives of *Lithops*. Previous studies on the function of similar foliar calcium oxalate formations show them to be translucent and to possess light scattering properties [16]. These properties suggest the presence of microcrystalline deposits within the below-ground epidermis of *L. aucampiae* leaves (Fig. 1(b) *iii*) may be associated with photosynthesis by scattering light within the below-ground region of the leaves, thus enriching the lower tissues with photons [16]. By increasing internal reflectance of incoming light from the above-ground plant mesophyll to the less illuminated below-ground photosynthetic tissues, we hypothesise that the presence of conical epidermal cells with polarised-light inference properties provide critical evidence of shade-specific adaptations present within the below-ground portions of *L. aucampiae* leaves with below-ground microcrystalline deposits in *Lithops* being analogous in function to the crystals frequently observed embedded within the epidermis and/or mesophyll of many shade-tolerant plant species. Cone-shaped epidermal cells were not observed on the epidermis and polarised light was not reflected in the above-ground region of the leaves (Figs. 1(b) *i,ii,vii,viii*).

Further evidence of the shade-tolerance capabilities of the below-ground region of *L. aucampiae* leaves lie in the increased concentration of chlorophyll *b* and reduced chlorophyll *a*∶*b* ratio of that region (Fig. 2a-d). Chlorophyll *a* occurs in all photosynthetic eukaryotes and is essential for commencement of photosynthesis. Chlorophyll *b* is not directly involved in photosynthetic processes but is important for broadening the wavelengths of light that can be absorbed by the organism and transferring ‘extra’ captured photons to chlorophyll *a* for use in photosynthesis [24]. In possessing a low chlorophyll *a*∶*b* ratio, a plant is effectively able to capture more photons across a broader range of wavelengths – an adaptation particularly important for plants growing in shaded environments. The above ground region appears to contain a marginally higher concentration of chlorophyll *a* and has a greater *a*∶*b* ratio than the below ground portion of the leaves, indicating greater photosynthetic capacity at higher light levels (Figs. 2*a, d*). This is confirmed by there being greater Ф_PSII_ (photosynthetic rate) at both high and low light levels in the above-ground region of the leaf compared to the below-ground portion. The lower Ф_PSII_ and greater NPQ in the lower parts of the leaf suggest that the effective ‘cost’ of increased chlorophyll *b* and reduced *a*∶*b* is borne through lower operational photosynthetic efficiency and greater dissipation of energy through NPQ (Figs. 2*e & f*).

These differences in regional photosynthetic function within the leaf structure may be enhanced by variation in light-blocking pigmentation. Pigment analysis identified numerous non-photosynthetic pigments contained in face and above-ground plant tissues. Flavonoids, of which several were identified to be present in the above-ground region (Table 1, Fig. 3), are known to filter UV radiation, and have been found to accumulate in the epidermal cells of other *Mesembryanthemum* species (Vogt *et al*. 1999). Unlike plants of the Caryophyllales, extracts obtained from *L. aucampiae* did not contain any compounds with UV absorbance within the range characteristic for betalain compounds. Flavonoid compounds, such as those detected here in *L. aucampiae,* generally accumulate in peripheral tissues exposed to high irradiance [25], therefore we might expect to see fewer of these compounds in the below-ground region. The brown pigmentation resulting from high accumulation of flavonoids is visibly reduced in below-ground leaf tissue compared to the above-ground leaf tissues (Fig. 1). Flavonoid pigments preferentially absorb green and blue light but reflect red wavelengths [26], thereby reducing the light energy available for photosynthesis, resulting in the lower NPQ values observed within the above-ground leaf tissues compared to the below-ground. This photo-protective biochemical mechanism is likely to be highly beneficial to wild-growing *L. aucampiae* which experience extremely high daily irradiance and would otherwise be highly susceptible to severe photo-damage and inhibition. The pigmentation of *L. aucampiae* is likely to also serve to protect it from herbivory by small mammals through camouflage.

Our metabolomic analysis confirmed previous findings that *L. aucampiae,* in common with other *Lithops* species [5], utilises CAM photosynthesis. PCA analysis demonstrated that *L. aucampiae* plants sampled pre-dawn had a very different metabolic profile to those sampled pre-dusk (SI). Many of the most highly discriminatory compounds identified through this analysis were organic acids, one of which was malate. The majority of these organic acids were in far greater abundance within plant tissues in the pre-dawn sampled plants when compared to those sampled pre-dusk (Table 2). Organic acids, malate in particular, are vital components of the CAM photosynthetic pathway, being produced overnight through CO_2_ fixation and stored in plant cell vacuoles until sunrise and the re-commencement of photosynthesis [24]. Plants that operate using CAM close their stomata during the day to reduce water loss through transpiration, inevitably inhibiting CO_2_ assimilation for use in photosynthesis. CAM plants, are able to mobilise and breakdown the vacuolar acids, such as those in Table 2, releasing carbon which is then utilised in the Calvin-Benson cycle [24]. Plants with these adaptations have relatively high tissue concentrations of organic acids (1.84 fold increase in the case of malate observed here, Table 2) when sampled pre-dawn compared to those sampled pre-dusk.

The simultaneous high-light, shade-tolerance strategies we have revealed within individual leaves of the xerophyte *L. aucampiae* are previously unreported in the plant kingdom and are, to our knowledge, unique to the Aizoaceae. We have shown the leaves of *L. aucampiae* are uniquely adapted to optimise photosynthesis under very different environments within the same structure: extreme high-irradiance in the AG region and shade conditions BG, and that these regions have sufficient physiological flexibility to respond to variable light conditions in a way that affords maximum protection against photo-inhibition and oxidative damage while maintaining optimal photosynthetic rates. This demonstrates unprecedented physiological flexibility in a xerophyte and is a step forward in our understanding of plant responses and adaptations to extreme environments.

## Supporting Information

Figure S1 **Principal component analysis (PCA) score scatter plot (a) of the metabolic fingerprinting data (direct injection mass spectrometry m/z values) obtained from** ***Lithops aucampiae*** **sampled at pre-dusk and pre-dawn.** The percent of the variation explained by each component is given. (**b**) molecular mass ions (m/z) differing the most between pre-dusk and pre-dawn samples. The molecular weight of malate is highlighted.
(PDF)

Methods S1 (DOCX)
