# Supplementary material for: Best of Both Worlds: Simultaneous High-Light and Shade-Tolerance Adaptations within Individual Leaves of the Living Stone Lithops aucampiae
Source: PLoS One. 2013 Oct 23;8(10):e75671. doi: 10.1371/journal.pone.0075671 (PMC3806800; doi:10.1371/journal.pone.0075671)
Supplement: Figure S1 — Principal component analysis (PCA) score scatter plot (a) of the metabolic fingerprinting data (direct injection mass spectrometry m/z values) obtained from Lithops aucampiae sampled at pre-dusk and pre-dawn. The percent of the variation explained by each component is given. (b) molecular mass ions (m/z) differing the most between pre-dusk and pre-dawn samples. The molecular weight of malate is highlighted. (PDF) [file pone.0075671.s001.pdf]

Best of both worlds: Simultaneous high-light and shade-tolerance adaptations within individual leaves of the living stone *Lithops aucampiae*

Katie J. Field, Rachel George, Brian Fearn, W. Paul Quick and Matthew P. Davey

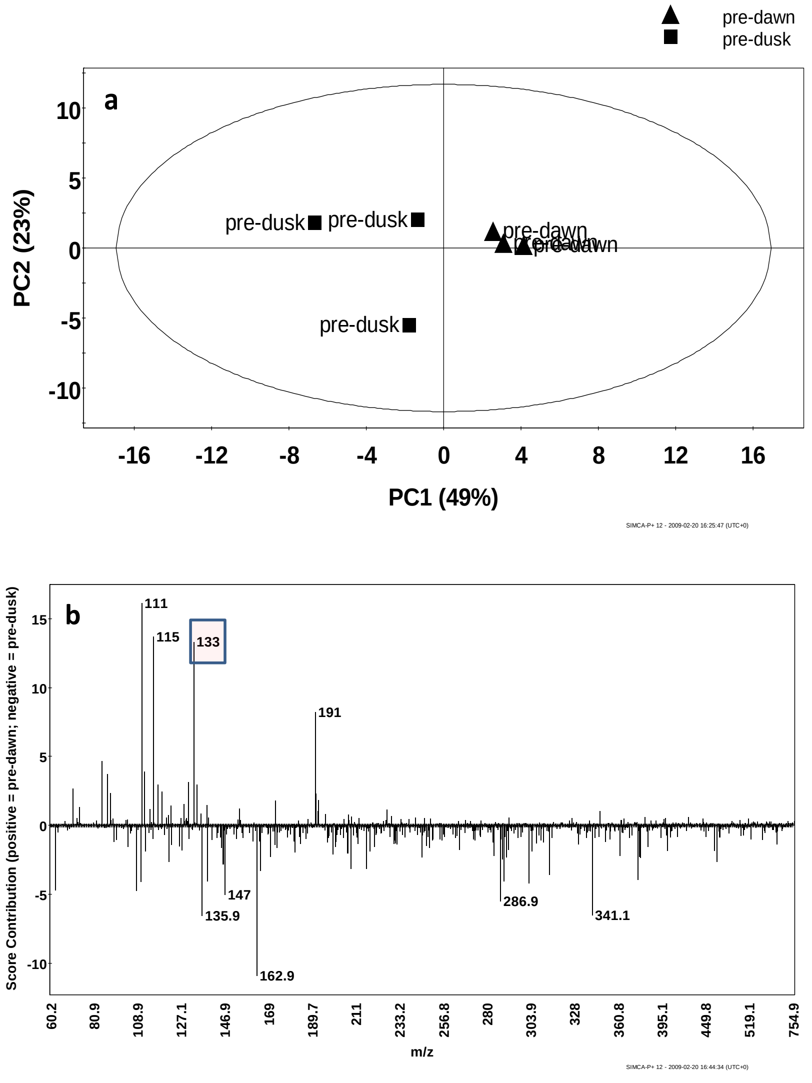

Figure S1. Principal component analysis (PCA) score scatter plot **(a)** of the metabolic fingerprinting data (direct injection mass spectrometry *m/z* values) obtained from *Lithops aucampiae* sampled at pre-dusk and pre-dawn. The percent of the variation explained by each component is given. **(b)** molecular mass ions (*m/z*) differing the most between pre-dusk and pre-dawn samples. The molecular weight of malate is highlighted.
