# Supplementary material for: Best of Both Worlds: Simultaneous High-Light and Shade-Tolerance Adaptations within Individual Leaves of the Living Stone Lithops aucampiae
Source: PLoS One. 2013 Oct 23;8(10):e75671. doi: 10.1371/journal.pone.0075671 (PMC3806800; doi:10.1371/journal.pone.0075671)
Supplement: Methods S1 — (DOCX) [file pone.0075671.s002.docx]

**Best of both worlds: Simultaneous high-light and shade-tolerance adaptations within individual leaves of the living stone *Lithops aucampiae***

***Katie J. Field, Rachel George_,_ Brian Fearn, W. Paul Quick and Matthew P. Davey***

**Supporting Information**

***Materials and Methods.***

*Plant material*

*Lithops aucampiae* plants were collected from the Abbey Brook *Lithops* UK national collection at Matlock, Derbyshire, UK where plants were grown in a glasshouse with Photosynthetically Active Radiation (PAR) ranging from 200 to 500 μmol m^2^ s^-1^ (http://www.abbeybrookcacti.com/). Plants were transferred into individual plug trays (2 x 2.5 cm) with a low-nutrient substrate (1:1 acid-washed silica sand: seed compost) and maintained under a day/night regime of 12/12hr at 20/15 ºC, 550 μmol m^2^ s^-1^ irradiance, 50 % Relative Humidity, 440 ppm CO_2_ in a controlled environment chamber (SGC2352/FM chamber, Sanyo-Gallenkamp, Japan) at the University of Sheffield, UK. These conditions mimicked conditions under which the plants were cultivated and therefore acclimated to. Plants were watered from above approximately every four weeks.

*Anatomy*

Longitudinal dissections were made by placing whole plants in foil cups, orientated so that slices would be cut in the desired plane, containing a fixing solution (Tissue-Tek O.C.T, Sakura finetek, Europe). Cups were placed in a cryostat chamber (Leica cm 1900) and left to freeze at -20 °C. The foil was removed and the block attached to a metal disk using fixing solution. Slices of the sample were made at a thickness of 60 nm. Slides were prepared using water to melt the frozen fixing solution around the plant tissue and viewed using an Olympus BX51 microscope (Olympus, Essex, UK). Fluorescence images were taken using UV excitation of 380-385 nm and an emission of 420 nm. Sections viewed under cross-polarised light microscope (Swift, Basingstoke, UK).

*Chlorophyll Fluorescence*

Chlorophyll fluorescence imaging was measured at time zero and after six weeks using a chlorophyll fluorescence imager (Technologica LTD, Colchester, UK) to quantitatively determine the activity, efficiency and spatial distribution of photosynthesis in *L. aucampiae.* *F_v_*/*F_m_* (maximum photosynthetic efficiency) was measured using plants that were dark-adapted for a minimum of 30 minutes prior to applying a photosystem-saturating 3000 photosynthetic photon flux density (PPFD) pulse for 200 ms. Actinic light levels of 100 and 500 µmol m^-1^ s^-2^, designed to replicate light conditions plants would experience throughout growth, were then applied prior to a further 3000 µmol m^-1^ s^-2^ PPFD pulse in order to obtain measures of the operational efficiency of photosystem II (Ф_PSII_) and the levels of light dissipated as heat (NPQ). The effect of rising PPFD was monitored in two plants grown at 550 µmol m^-1^ s^-2^ for six weeks before placing them in the chlorophyll fluorescence imager and incrementally increasing the actinic light level from 100 to 1000 µmol m^-1^ s^-2^ over a period of 15 minutes.

*Quantification of chlorophyll and carotenoids*

Tissue samples were taken from the face and both leaf surfaces, in both the above- and below-ground regions (Fig. 1) using a 2mm diameter tissue corer. Two cores were taken from each area to a depth of 3 mm providing 60 mm^3^ of tissue sample. Tissue samples were placed in 2 mL plastic Eppendorf tubes, immediately frozen by immersion liquid nitrogen and homogenized using a small pestle. 1.5ml of 96 % ethanol was added to each tube prior to heating (70°C) for 15 minutes. The green ethanol supernatant was decanted into 5 ml test tubes and stored in the dark. A further 1.5 mL of 96 % ethanol was added to the leaf sample pellet and heated to 70 °C as above. The previous two steps were repeated until the leaf tissue was completely colourless. The absorbance of this decanted ethanol was measured at 665 nm, 649 nm and 470 nm using a spectrophotometer (Lambda 40 UV/VIS, Perkin Elmer, Massachusetts, USA). Concentrations of chlorophyll *a* and *b* and carotenoids on a leaf volume basis were calculated according to reference ([Wellburn 1994](#_ENREF_18)).

*Metabolomics*

For HPLC analysis of pigmentation in *L. aucampiae*, three samples per plant were taken from the face epidermis or abaxial epidermis (both brown and green tissue) of six plants. Bi-phasic metabolite extracts were prepared directly from the frozen plant tissue through homogenization, addition of a cold extraction medium comprising chloroform, methanol and water, and the resultant phases were separated. The aqueous and organic phases of the *L. aucampiae* samples were analysed by high performance liquid chromatography (HPLC) (Hewlett Packard Series 1090 liquid chromatography system). 50 μl extracts were resolved using ethyl acetate with 0.1 % triethylamine (solvent A) and 90 % methyl cyanide with 10 % H_2_0 and 0.1 % triethylamine (solvent B); with a gradient of increasing solvent A such that initial A:B (0:100 v/v); 10 min, (20:80); 20 min, (90:10); 30min, (90:10); 32 min, (0:100); 35 min, (0:100); at a flow rate of 1ml min^-1^. The extracts were monitored for absorbance between 250 nm and 600 nm.

Broad-spectrum metabolite fingerprinting was carried out to confirm CAM within *L. aucampiae* by using Direct Injection Mass Spectrometry (DIMS). Briefly, six plants were removed from the high light growth chamber, three at 05:30 hours (pre-dawn) and three at 17:30 hours (pre-dusk) and were immediately frozen in liquid nitrogen. The entire frozen plants were then homogenized and a 100mg tissue sample taken. From this, metabolites were extracted as per the methods above. The aqueous phases of the extract were directly injected into a LCT mass spectrometer (Waters Ltd. Manchester, UK) using a MassLynx V.4.0 data system in negative ionisation mode. Metabolite profiles were compared by unsupervised Principal Component Analysis (PCA) using Simca-P+V12.0 (Umetrics, Sweden) as is appropriate with such large, multivariate datasets. The binned mass numbers were used as the primary variable and sample time (i.e. pre-dawn or pre-dusk) as observational variables. R^2^ values were obtained through analysis for each primary variable and their contribution to the discriminating observation variable in the PCA, giving us direct quantification of each mass number’s relative contribution to variation observed in the PCA plot. *m/z* values identified as highly discriminatory (with a R^2^ value of close to 1.0) were identified using the comprehensive online databases KEGG (<http://genotome.jp/kegg/>) and Aracyc (<http://metacyc.org/>)
